# The comorbidity burden of patients with cluster headache: a population-based study

**DOI:** 10.1186/s10194-017-0785-3

**Published:** 2017-07-24

**Authors:** Shivang Joshi, Paul Rizzoli, Elizabeth Loder

**Affiliations:** 1 Clinical Pharmacy Practice, MCPHS University College of Pharmacy, Worcester, USA; 2 Community Neuroscience Services, Westborough, MA 01581 USA; 3 Harvard Medical School, Boston, USA; 4 Graham Headache Center, Brigham and Women’s Faulkner Hospitals, Boston, MA 02130 USA; 5 Division of Headache, Department of Neurology, Brigham and Women’s Faulkner Hospitals, Boston, MA 02130 USA

**Keywords:** Cluster headache, Comorbidity, Diagnostic delay, Misdiagnosis, Healthcare utilization

## Abstract

**Background:**

Evidence is limited regarding the comorbidity burden of patients with cluster headache (CH). We aimed to characterize comorbid conditions in a cohort of CH patients diagnosed by headache experts, using electronic health record information from the Partners Research Patient Data Registry (RPDR).

**Methods:**

We identified and reviewed the charts of unique patients diagnosed by headache specialists over an 11-year period, and a set of matched controls. Patients were categorized as having Definite, Unconfirmed or no CH. We calculated the prevalence of and tested for statistically significant differences of selected comorbid conditions in these populations.

**Results:**

An RPDR query identified 170 patients with a free text or ICD diagnosis of cluster headache. 15 records belonging to Partners employees were excluded. 75 patients met diagnostic criteria for CH (Definite CH). 22 had headaches with some features of CH but the diagnosis was uncertain (Unconfirmed CH). In 58 the diagnosis was determined to be inaccurate due to data entry errors. Patients with Definite CH had an average age of 43.4 years; 80% were male. The average time from CH onset to diagnosis was 12.7 years (range 1–51). The average number of yearly emergency department and outpatient visits for the group of Definite CH patients was 4.5 and 25.4, respectively, compared with 1.1 and 6.9 in controls. Of the 55 examined conditions, four were statistically significantly less common in patients with definite CH compared with controls (diabetes, musculoskeletal/orthopaedic problems, “other gastrointestinal diagnoses” and skin conditions) and four were statistically significantly more common (smoking, depression, dental disorders and deviated septum).

**Conclusions:**

In this large population-based study, we identified a surprisingly small number of patients who met strict diagnostic criteria for CH. In these patients, however, we identified a distinct pattern of selected comorbidities. The pattern is somewhat but not entirely consistent with that of the “classic” CH patient depicted in the medical literature. CH patients are frequently diagnosed with sinus or dental problems. Many experience substantial delay in receiving a diagnosis. These things may in part explain the high frequency of medical visits in this population. It is difficult to distinguish conditions that are genuinely comorbid with CH from those that reflect misdiagnoses or medical scrutiny of patients in frequent contact with the healthcare system.

## Background

Cluster headache is a relatively uncommon primary headache disorder that is one of the trigeminal autonomic cephalgias. Its lifetime prevalence is estimated to be 124 per 100,000 persons with a one-year prevalence of 53 per million [1, 2]. Cluster headache is considered to be among the most severe forms of pain. Patients with cluster headache experience substantial decrements in quality of life and have many other health-related burdens [3].

Comorbidity is defined as the presence of separate illnesses in the same patient at a frequency greater than would be expected by chance [3, 4]. Clinical experience and previous research, summarized in Table 1, suggest that CH patients have a higher prevalence than the general population of a number of comorbidities, including such things as coronary artery disease, head trauma, peptic ulcer disease, and alcohol and tobacco use. However, previous studies were small, did not always include representative samples of patients with CH, and had other methodological limitations. For these reasons, using information from a specialty headache clinic located in a large academic medical center, we aimed to study the comorbidity burden of patients with CH.

**Table 1:** Selected previous studies of comorbidity in cluster headache

Author, Year	Population, setting	Design	Comorbidities	Comments
Ferrari, 2013 [15]	200 consecutive male and female CH patients from Italian headache clinic	Cross-sectional survey study	60% were current, 21% former, 19% never smokers.	No change in headache noted in those who had stopped smoking.
Kudrow, 1976 [16]	140 male and female CH patients vs. controls from a California headache clinic and healthy outpatients	Cross-sectional chart review	Men with CH had a statistically significantly higher prevalence of peptic ulcer disease compared with controls. No increased risk of coronary artery disease was demonstrated.	
Lambru, 2010 [17]	200 male CH patients and 200 migraine controls from Italian headache clinic	Cross-sectional chart review	Prevalence of traumatic head injuries 38.5% in those with CH vs. 23% in controls (OR 2.0 (95% CI 1.3 to 4.9). Prevalence of alcohol use was 74.5% and cigarette smoking 75% in those with CH.	Only males included in this study.
Liang, 2013 [18]	673 male and female CH patients from a Taiwanese National Health Database	Retrospective cohort study with 2.5 year median follow-up duration	3.6% developed depression over study period. Adjusted HR 5.6% vs. controls but not different from those with migraine; number of bouts/year of CH a risk factor for depression.	Study limited to patients diagnosed by neurologist and prescribed standard CH drugs; excluding those with previous psychiatric diagnoses (104 of original 777; 13%).
Pietrini, 2005 [19]	60 consecutive male and female CH patients seen at an Italian headache center	Cross-sectional, based on study examination	35% had hypertension, defined as blood pressure ≥ 140/90 on average of 3 blood pressure readings.	The authors concluded that the prevalence of hypertension in this group was within expected range given age and sex.
Robbins, 2012 [20]	49 consecutive male and female CH patients seen in a New York headache clinic over a 3.5 year period	Cross-sectional chart review	Prevalence of depression (PHQ ≥ 1 0) was 6.3% in episodic CH, 11.8% in chronic CH; Anxiety (GAD-7 ≥ 10) prevalence was 15.6% in episodic CH and 11.8% in chronic CH. Prevalence of hypertension was 14%, current or former cigarette smoking was 65.3% and GERD was 8%.	
Rossi, 2012 [21]	210 consecutive male and female CH patients from two Italian headache centers	Cross-sectional interview and anonymous survey data	92.5% of male CH patients and 85.4% of female CH patients reported current or past use of tobacco, statistically significantly higher than prevalence in general population.	Self reported data on substance use.
Rozen, 2012 [22]	1134 US male and female CH patients responding to an internet survey	Cross-sectional survey (internet)	Prevalence of depression was 24%, suicidal ideation was reported by 55%, sleep apnea by14%, restless leg syndrome by 11%, asthma by 9%. 73% were current or former smokers; 51% had been smoking at the time CH began. 65% used alcohol but only 3% reported a history of alcohol abuse. The prevalence of coronary problems was low: 1% had a history of myocardial infarction, 0.3% bypass surgery and 1% stent placement. Peptic ulcer disease was reported by 5%, diabetes by 3% and epilepsy by 1%.	Unlikely to be a representative sample of patients with CH due to self-selected nature of participants. Diagnosis not medically verified. Respondents compared with the US population in general rather than matched controls. Data should be interpreted cautiously.
Van Alboom, 2009 [9]	85 male and female CH patients from 4 Belgian neurology clinics	Cross-sectional chart review	45% had been diagnosed with migraine, 23% with sinusitis, tooth/jaw problems 23%, trigeminal neuralgia 16%.	Average diagnostic delay was reported to be 44 months.
Voiticovschi –Iosob, 2014 [23]	144 male and female Italian and Eastern European patients with episodic cluster headache	Cross-sectional (diagnostic interview and survey)	16% of CH patients had previously been diagnosed with sinusitis; 4.2% with dental disorders.	Average delay between onset of symptoms and diagnosis in this sample was 5.3 years. 10.4% had consulted an otolaryngologist and 2.8% a dentist.
Xie, 2013 [24]	26 male and female CH patients identified by survey in tertiary Chinese headache clinic	Cross-sectional (diagnostic interview)	14/26 (54%) were current smokers, 19.2% former and 26.9% nonsmokers.	The reported prevalence of smoking in those with CH is in line with that in the general population of Chinese men. According to 2010 data, 53% of Chinese men and 2.4% of women are smokers.
Zidverc-Trajkovic, 2011 [8]	130 consecutive male and female CH patients and 982 with migraines in a specialty headache clinic	Cross-sectional chart review	Prevalence of anxiety or depression in CH was 4.6%, chronic sinusitis 3.6% diabetes mellitus 3.8%.	

*Abbreviations*: *CH* cluster headache, *GAD-7* generalized anxiety disorder 7-item scale, *GERD* gastroesophageal reflux disease, *HR* hazard ratio, *ICHD* International classification of headache disorders, *OR* odds ratio, *PHQ-9* patient health questionnaire 9-item scale

## Methods

The Partners Healthcare Institutional Review Board approved the study. We used the Partners Research Patient Data Registry (RPDR) to identify unique patients with CH. The RPDR is a relational database containing clinical and administrative information on millions of patients seen within the Partners Healthcare System [5]. Partners Health Care is a not-for-profit, integrated health care system in Boston, Massachusetts that provides care for roughly 50% of the population in the Boston metropolitan area. Partners Health Care includes community and specialty hospitals, a managed care organization, a physician network, community health centers, home care and other health related services. During the period of this study, Partners Healthcare used a proprietary electronic medical record system known as the Longitudinal Medical Record (LMR). Physicians could directly dictate or enter typed information into the medical record. Physicians recorded International Classification of Diseases, version 9 (ICD-9) diagnoses on a billing sheet by circling or checking a list of common diagnoses. Physicians could also hand-write an ICD-9 code on this sheet. This diagnostic information was entered into the electronic billing system by billing assistants and subsequently imported into RPDR.

We searched for patients who had received a diagnosis of cluster headache during a 10 year period between 2002 and 2012 from one of nine headache specialists known to be practicing within the Partners system during this period. The search was limited to patients diagnosed by headache specialists because the accuracy of cluster headache diagnoses by non-specialists is known to be low [6].

We identified medical record numbers of unique patients seen between 1/1/2002 and 12/31/2012 who had received a diagnosis of cluster headache. Patients diagnosed with cluster headache were identified in two ways. First, we searched for patients for whom an International Classification of Diseases (ICD) code for cluster headache had ever been recorded. For example, the ICD-9 code for episodic cluster headache is 339.01. Second, we searched for charts in which relevant terms (“Episodic Cluster Headache”, “Chronic Cluster Headache” or “Cluster Headache”) appeared in the free text problem list, as a diagnosis not associated with an ICD code, or elsewhere in the medical record. Our final list consisted of patients who met either of these criteria, with duplicate records removed. For reasons of privacy, we did not include records belonging to patients who were employees of Partners Healthcare. Charts that appeared to be inaccurately coded were also removed. For example, patients who had received a diagnostic code for CH but who were described in the notes as having migraine were eliminated based on the assumption that a data entry error had occurred.

Two researchers independently examined each medical record and attempted to validate the diagnosis of cluster headache by locating information required to make a diagnosis of cluster headache. Information from each eligible patient’s record indicating the physician considered him or her to have CH was considered the gold standard for the presence of CH. Patients were categorized as having “Definite cluster headache” if the medical record contained sufficient information to make a diagnosis of cluster headache according to ICHD-2 criteria [1]. We did not distinguish between episodic and chronic forms of the disorder. Patients were categorized as having “Unconfirmed cluster headache” if information in the medical record indicated they met some criteria for cluster headache, but information was insufficient to make a Definite diagnosis according to ICHD criteria.

Using structured RPDR query methods, we identified two age and sex matched controls without cluster headache for each patient in the group of Definite CH patients. RPDR allows researchers to define criteria for the selection of healthy controls, and to choose the number of controls for each case patient. Using an algorithm, patients matching these criteria, but without the disease in question, are randomly selected and their medical record numbers are returned to the researcher, who is then able to retrieve the full medical record.

The medical records of patients with Definite and Unconfirmed cluster headache, as well as controls, were then hand-searched by one author (SJ), who abstracted information on headache characteristics, age at onset and age at diagnosis of CH, as well as relevant comorbidities using a standardized data abstraction form. A second author (EL) reviewed a subset of medical records to verify the accuracy of data abstraction. Differences were resolved through consensus. We recorded all comorbid disorders identified in patient records, resulting in the list of 56 conditions listed in Table 2. The same methods were used to search for comorbidities in the charts of CH patients and those of controls, namely one authors (SJ) used the RPDR Data Query tool to search for diagnoses of these 56 conditions. The Query tool allows researchers to use the Notes Search function, which searches in all ambulatory notes and clinical reports for a particular patient for specific diagnoses or terms. A diagnosis of alcohol abuse was inferred if the patient was recorded as ever participating in an alcohol abuse program or if listed as a medical condition by the physician on a problem list or appeared as a diagnosis in the medical record. Similarly, patients were categorized as cigarette, cigar or other substance users if the patient was recorded as ever using those substances.

**Table 2:** Demographic characteristics and prevalence of comorbid conditions in patients with definite and unconfirmed CH and controls

	Definite *n* = 75	Control *n* = 152	Unconfirmed *n* = 22	
Mean age in years (range)	43.4 (20–74)		44 (20–74)	
Proportion of males	80%	80%	45%	
Comorbidity	%	%	%	*p* value definite v controls Pearson chi-square
Attention deficit disorder	4	2	0	0.4
Anxiety	7	8	18	0.7
Arthritis/Rheumatologic condition	15	11	14	0.4
Asthma	7	7	0	1
Back pain/Spine condition	17	14	14	0.56
Celiac disease	1	0	0	0.32
Cerebrovascular disease	1	1	0	1
Cigar smoking	4	1	0	0.17
Cigarette smoking	64	32	31	0.00077***
Congenital disease	0	3	0	0.08
COPD	1	1	0	1
Cardiovascular disease	15	16	36	0.85
Dental/TMJ	4	0	5	0.04*
Depression	17	7	23	0.03*
Deviated septum	7	1	5	0.03*
Diabetes	0	9	5	0.002**
Divorce	13	7	14	0.16
Endocrine	8	3	9	0.12
Alcohol abuse	17	8	5	0.054
Alcohol moderate	1	3	0	0.31
Fibromyalgia	5	1	0	0.1
GERD	8	10	9	0.62
GI procedures	7	8	0	0.79
Glaucoma/Ocular	3	3	5	1
Head trauma	5	1	9	0.1
Hematologic	3	7	14	0.19
Hyperlipidemia	25	25	32	1
Hypertension	15	22	18	0.2
Irritable bowel syndrome	4	1	14	0.17
Infectious	5	4	9	0.73
Malignancy	9	16	14	0.13
Marijuana	8	4	9	0.23
Musculoskeletal/Ortho	7	16	5	0.046*
Nephrolithiasis	7	2	0	0.08
Ob/Gyn	4	5	0	0.73
Obesity	3	7	9	0.19
Other GI	9	24	5	0.004**
Other Headache	5	3	5	0.47
Other Neurologic	4	7	9	0.35
Other Pain, e.g. fibromyalgia	3	5	0	0.47
Other Psychiatric disorder	7	5	14	0.55
Other sleep	3	5	5	0.47
Other Substance abuse	3	4	9	0.7
Peptic ulcer disease	4	1	9	0.17
Renal	1	3	0	0.31
Restless legs	0	0	0	1
Seizure disorder	5	5	5	1
Sinus problems	5	8	9	0.39
Skin conditions	4	19	5	0.0008***
Sleep apnea	9	5	5	0.27
Suicide attempt	0	1	0	0.32
Tendinitis	7	3	5	0.19
Trigeminal neuralgia	0	0	0	1
Urologic	5	10	5	0.18
Vascular malformation	1	0	0	0.32
Violent trauma	0	2	0	0.16

*Abbreviations*: *ADD* attention deficit disorder, *COPD* chronic obstructive pulmonary disease, *GI* gastrointestinal, *Ob/gyn* obstetrical or gynecological diagnoses

*significant at <0.05, **significant at <0.01, ***significant at <0.001

We also performed queries in RPDR to determine demographic characteristics and other information such as the average yearly number of emergency and outpatient visits for the entire CH group (Definite and Unconfirmed), the age and sex-matched control group, and the entire population of patients in RPDR, the latter to help put results for the CH population in context.

### Analysis

All data were analyzed using SPSS statistical software (IBM Corp. Released 2012. IBM SPSS Statistics for Windows, Version 21.0. Armonk, NY: IBM Corp.) We calculated Pearson’s chi-square statistics for differences in proportions and considered a *p* value <0.05 to be significant. We also distinguish between comparisons for which the *p* value is <0.01 and <0.001, as a way of allowing readers to account for multiple comparisons.

## Results

**Fig. 1:**
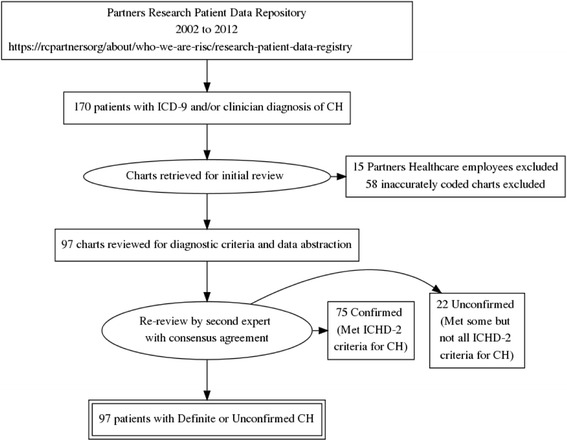
Flow of patients through the study. Abbreviations: ICD = International Classification of Diseases; ICHD-2 = International Classification of Headache Disorders, 2nd edition; CH = Cluster headache

Figure 1 shows the flow of patients through the study. The initial search from 12/1/2002 to 6/30/2012 among nine headache specialists identified a total of 170 patients who had received an ICD-9 diagnosis of CH and/or had a free text diagnosis of CH recorded by the physician or included in the problem list. 116 of these patients were identified by ICD-9 codes alone. After removal of patients who were employees of the Partners Healthcare system, 155 patients remained. Following exclusion of 58 inaccurately coded charts, a total of 97 patients remained in the final analysis. After chart review and data abstraction by 2 experts, 75 patients were determined to clearly meet criteria for cluster headache (Definite CH). For these 75 patients, RPDR identified 152 age and sex-matched controls. 22 patients had headaches with some features of CH but the diagnosis could not be confirmed from information contained in the medical record (Unconfirmed CH). The main reason that patients were included in the Unconfirmed rather than the Definite CH group was duration of headache over 3 h.

**Fig. 2:**
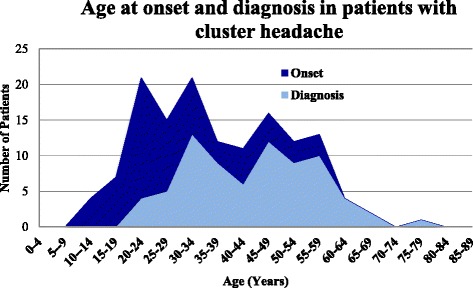
Age at onset and diagnosis in 75 patients with definite cluster headache

**Fig. 3:**
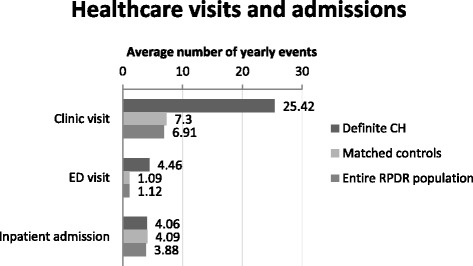
The average number of yearly emergency department (ED) and clinic visits was higher for CH patients compared with the entire RPDR population or controls

Figure 2 illustrates the distribution of patients with Definite CH by self-reported age at onset of CH in comparison with age at the time of CH diagnosis. The average time from first appearance of symptoms to diagnosis was 12.7 years (range 1 to 51). Figure 3 shows the average number of yearly inpatient, clinic and emergency department visits for patients with Definite CH, age and sex-matched controls and (to provide context) the entire population of patients in the RPDR. CH patients had a higher average number of emergency department and clinic visits compared to either controls or the entire RPDR population.

Table 2 shows the demographic characteristics and comorbidity burden of patients with Definite CH compared to those with Unconfirmed CH and matched controls. The average age of the Definite CH group was similar to that of the Unconfirmed group (43.4 v 44 years), but the sex ratio was notably different. 60/75 (80%) of patients with Definite CH were men, compared to just 10/22 (45%) with Unconfirmed CH.

The prevalence of eight of the 56 studied comorbidities was statistically significantly different in patients with Definite CH compared with age and sex-matched controls. Specifically, patients with Definite CH had a higher prevalence of diagnoses of cigarette smoking, deviated septum, dental/TMJ problems and depression than controls, and a lower prevalence of skin problems, diabetes, musculoskeletal complaints or “other gastrointestinal” problems.

## Discussion

In this large population-based study, we identified a surprisingly small number of patients who met strict criteria for CH, but a larger number who were considered by expert clinicians to have a CH disorder. This suggests that the sensitivity of existing diagnostic criteria in clinical practice is low. However, in patients diagnosed by expert headache clinicians with CH over an 11 year period, we did identify a distinct pattern of selected comorbidities compared with age and sex-matched controls. The pattern of patient characteristics and comorbidities that emerged from our study is somewhat but not entirely consistent with that of the “classic” CH patient depicted in the medical literature. Our study does not suggest that CH patients are more likely to have cardiovascular disease, or to use or abuse alcohol in comparison with non-CH controls. This is not necessarily inconsistent with prior studies, which often looked at alcohol consumption and use; even if CH patients are more likely to drink alcohol than non-CH sufferers, it is not clear they are more likely to have alcohol abuse. In our study CH patients were less likely to have diabetes than controls. The majority of patients diagnosed with cluster headache in this population were men and they were more likely to have received diagnoses of depression, and of deviated septum or dental/temporomandibular joint problems, perhaps reflecting previous inaccurate diagnoses of the cause of their unilateral head pain rather than true comorbidities.

In fact it is difficult to distinguish between conditions that are comorbid with CH and those that represent misdiagnoses of the disorder or selection bias due to the high number of healthcare visits that CH patients have. This increases the likelihood that latent medical problems will be diagnosed during evaluation and testing, a phenomenon known as “Berkson’s bias” [7]. CH patients are frequently diagnosed with sinus or dental problems, and many experience substantial delay in receiving a diagnosis [8–10]. These things may in part explain the high frequency of medical visits in this population. Nonetheless, caution should be used in interpreting our results, since it is not possible to distinguish conditions that are genuinely comorbid with CH from those that reflect misdiagnoses or result from increased medical scrutiny of patients in frequent contact with the healthcare system. Several of the comorbidities we identified, particularly sinus or dental problems, are very likely to reflect misdiagnoses of CH rather than true comorbid conditions.

Although patients with Definite CH were statistically significantly more likely to have a history of cigarette smoking than controls, this was not true for cardiovascular conditions or alcohol use disorders. The prevalence of diabetes was significantly lower in patients with CH than in matched controls. This raises the question of whether diabetes might protect against the development or expression of CH, something that could be explored in future studies. Notably, CH patients had a statistically significantly elevated prevalence of diagnoses of deviated septum and dental or temporomandibular problems, which might represent previous inaccurate diagnoses of their unilateral head pain.

Our data are consistent with previous findings of a long delay between the appearance of CH symptoms and receipt of a correct diagnosis [11, 12]. Our findings also quantify the increased use of healthcare among patients with CH. A previous study from Denmark found that 43.5% of CH patients had consulted a general practitioner during the previous year, compared with 9.2% of the general public. 43.5% had consulted a specialist, compared with 3.3% of the general public [3] In our study, CH patients had roughly 3 times the number of outpatient and 4 times the number of emergency department visits in an average year, indicating a high medical burden. It is possible that some of these visits represent attempts to obtain an accurate diagnosis or effective treatment for CH. This suggests that healthcare payers could realize important costs savings through improved recognition and treatment of CH.

The majority of patients in the Unconfirmed CH group were female. This is in contrast to the predominance of males in the Definite CH group. In this subgroup a major reason patients did not meet criteria for Definite CH was duration of CH longer than the three-hour maximum allowed by diagnostic criteria. A longer duration of attacks in women compared with men who have CH was not noted in a previous study that examined sex-specific attack differences [13]. Thus, it is possible, even likely that many patients in the Unconfirmed group did not have cluster headache but instead had migraine with prominent autonomic symptoms. The sex distribution would support this hypothesis. On the other hand, the clinicians making the diagnosis of CH were experienced headache experts, who presumably considered this possibility but still felt that the headaches were more likely to be CH.

It is interesting to note that the pattern of comorbidities and diagnostic delay was very similar in the Definite and Unconfirmed groups. These similarities support the view that these groups suffer from a single disorder. The ICHD criteria represent a compromise between the clinical need for sensitive criteria and the research need for specificity. It is thus not surprising that the sensitivity of the criteria is somewhat lower than ideal for clinical purposes. Clinicians should bear in mind that in clinical practice, strict adherence to ICHD criteria would result in missing patients who likely do have CH and would benefit from treatment. This is especially true for women with the disorder.

## Conclusions

Our findings provide high quality information in a representative sample of patients with expert-confirmed and carefully validated cluster headache diagnoses seen in a large academic medical system over the course of a decade. Strengths of this study include careful validation of CH diagnosis, an electronic medical record with semi-standardized data entry that allowed thorough characterization of the natural history and clinical course of patients, as well as high quality, complete information on comorbidities in our patient population and age and sex matched controls. Our study also has a number of limitations. We performed multiple comparisons, which increases the chance that some findings are false positives. Because of this our findings should be viewed as descriptive and hypothesis-generating. In most cases, comorbidities were diagnosed clinically rather than using structured diagnostic criteria. We used the second version of the ICHD criteria for this study rather than the current 3-beta version [14]. However, the only change to the diagnostic criteria in the latest version was the addition of a “sensation of ear fullness” or “forehead and facial flushing” to the list of ipsilateral autonomic symptoms or signs. These symptoms were not mentioned in any of the clinical notes we reviewed, so these minor changes are unlikely to have any material effect on our findings or conclusions.

## Abbreviations

CH: Cluster headache
EHR: Electronic health record
RPDR: Research patient data registry
